# An Indigenous physician’s response to the settler physician perspective on Indigenous health, truth, and reconciliation

**Published:** 2018-11-12

**Authors:** Marcia Anderson

**Affiliations:** 1 Indigenous Academic Affairs, Ongomiizwin Indigenous Institute of Health and Healing, Rady Faculty of Health Sciences, University of Manitoba, Manitoba, Canada

Jaworsky’s recent article “A settler physician perspective on Indigenous health, truth, and reconciliation” provides excellent suggestions for her peers on allyship.^1^ The Anti-Oppression Network defines allyship not as an identity, but as the “active, consistent and arduous practice of unlearning and re-evaluating, in which a person in a position of privilege and power seeks to operate in solidarity with a marginalized group.”^2^ Jaworsky demonstrates this by acknowledging the Indigenous people and medical educators that she has learned from, by referencing Indigenous scholars, and by amplifying the voices of Indigenous people. She begins the piece by locating herself and her position of privilege, the same way I would introduce myself according to our protocols in the language, by my nation and community links. I am a Cree-Anishinaabe woman who grew up in the North End of Winnipeg with roots to Norway House Cree Nation and Peguis First Nation. The Cree and Anishinaabe lines of my family have always been on Turtle Island.

Jaworsky provides four separate suggestions for her peer settler physicians, acknowledging that they are not comprehensive or sufficient and cannot replace the critical dialogue led by Indigenous physicians. If I were to offer a critique, it would be that, as physicians, we too often rush to the action steps without considering the how, and in the case of reconciliation the how is particularly critical. She suggests, for example, that we honour Indigenous expertise, by including Indigenous Health Experts and Knowledge Keepers on our medical education teams, but she needs to further elaborate and build upon this concept.

The Truth and Reconciliation Commission of Canada (TRC) has provided us with a roadmap of how we should move forward in resetting the relationship between Indigenous people and Settler Canada.^3^ The first principle of reconciliation is that the United Nations Declaration on the Rights of Indigenous Peoples (UNDRIP) is the framework for reconciliation at all levels and across all sectors of society. It is thus imperative that Settler physicians and medical learners have a working knowledge of UNDRIP, its key themes, and their responsibilities in a rights-based approach to reconciliation. Two of the relevant major themes are the right to be self-determining, which is related to Jaworsky’s point not just of honouring Indigenous expertise but actually making space for Indigenous leadership, and secondly, the right to be free from racism.

As we work towards understanding and acknowledging the truth before reconciliation, we need to be deliberate about identifying and naming the ways racism operates in our health care and medical education systems. Jaworsky has started this with the example of Brian Sinclair. In the subsequent paragraph on paternalism and cultural superiority within medicine this could be appropriately identified as epistemic racism: the privileging of one knowledge system over another. Sustained critical reflection on how evidence-based medicine can conflict with Indigenous peoples’ rights to receive equitable health care and to use traditional medicines as they decide best for them will be an important part of moving forward together in a new way.

Discussions about reconciliation are useful only insomuch as they result in improved health and social outcomes for Indigenous peoples. The critical self-reflection called for in the paper provides an important foundation for action. However, this must be translated into individual and collection action. Only when Indigenous peoples are receiving equitable, high quality care and we are making progress on closing the persistent gaps in Indigenous health should we consider the job well done.
